# Revealing the boon and bane of South Africa’s disaster management legislation during COVID-19

**DOI:** 10.4102/jamba.v16i1.1689

**Published:** 2024-10-31

**Authors:** Livhuwani D. Nemakonde, Olivia Kunguma

**Affiliations:** 1 Department of Geography and Environmental Studies, North-West University, Potchefstroom, South Africa; 2 Disaster Management Training and Education Centre for Africa, Faculty of Natural and Agricultural Sciences, University of the Free State, Bloemfontein, South Africa

**Keywords:** COVID-19, Sars-CoV2, disaster legislation, policy, South Africa, pandemic

## Abstract

Legislation provides a framework for effective and coordinated disaster preparedness and response. This article evaluates the strengths and shortcomings of South Africa’s disaster risk management (DRM) legislation in guiding the response to the COVID-19 pandemic. Since its promulgation in 2002, South Africa’s DRM legislation has been hailed as one of the most progressive legislations globally. However, the severe respiratory syndrome coronavirus 2, commonly known as the COVID-19 pandemic, exposed inadequacies in most existing DRM legislation worldwide, including in South Africa. This led to an inadequate response to the pandemic. A content-based literature review was conducted. Forty-nine peer-reviewed articles, reports, op-eds and newspaper articles were included in the review. The review highlighted significant inadequacies of South African DRM legislation, including the placement of the National Disaster Management Centre and the establishment of new structures for COVID-19 response. Based on the findings of this study, disaster managers, as the primary implementers of the disaster management legislation, must motivate the constant review of the disaster management legislation as a way of mitigating social, economic, political and environmental impacts of disasters, which emanate from the inadequacies existing in the disaster legislation.

**Contribution:**

The study’s findings contribute to the effective management of the disaster management fraternity by suggesting amendment of the legislation based on the experience during the pandemic. The recommendations made to disaster managers will assist with responding appropriately to future pandemics and other disasters.

## Introduction

Since the promulgation of the *Disaster Management Act* (Number 57 of 2002 as Amended, No. 16 of 2015) (DMA), this legislation has been glorified and hailed as one of the most comprehensive and progressive Disaster Risk Management (DRM) legislation. Subsequently the concept of Disaster Risk Reduction (DRR) was introduced in early 1990 (Kunguma 2022; Pelling & Holloway 2006; Vermaak & Van Niekerk 2004). As Van Niekerk (2014) pointed out:

[*T*]he promulgation of the South African DMA and the National Disaster Management Policy Framework of 2005 (NDMF) placed South Africa at the international forefront by integrating disaster risk reduction into all spheres of Government through a decentralised approach. (p. 858)

Pelling and Holloway (2006:4) opine that South Africa’s DMA was applauded internationally as a path-breaking example of national legislation that promotes DRR. As a result, the act has generated particular interest as an example of international best practice, especially in profiling the role of legislation in driving the integration of DRR action across multiple sectors and disciplines (NDMC 2010; Kunguma 2022; Pelling & Holloway 2006). However, some of the powers exercised by the Executive branch of South Africa’s government during the COVID-19 response under the DMA put to the test the strength and resilience of South Africa’s constitutional democracy (Padayachee et al. 2020). These included but were not limited to the lockdowns, closing of schools and businesses, limiting travel and physical distancing (Kotzé 2022).

With this, in hindsight, it is important to evaluate the strengths and limitations of South Africa’s disaster management system as provided for in the DRM legislation in guiding response to pandemics such as COVID-19. This is important because the DMA framed the country’s response to the pandemic after the state of disaster declaration. The pandemic brought on significant social, economic, political and environmental difficulties. Some of the difficulties are still felt post-pandemic (Padayachee et al. 2020). Despite the difficulties felt, the pandemic presented opportunities for policy change and learning (Crow et al. 2023; Taylor et al. 2022).

This article evaluates the strengths and limitations of South Africa’s DRM legislation in guiding the response to the COVID-19 pandemic. In the process, the articles also outlines the importance of legislation in addressing disaster risk and outlines a brief history of the South African DRM legislation. This is followed by an outline of the research methods applied in the study. The study’s findings are then presented and discussed before conclusions are drawn.

### Importance of disaster legislation in reducing the risk of disasters

The first ‘Priority for Action’ of the Sendai Framework for Disaster Risk Reduction (2015–2030) (SFDRR) is ‘strengthening disaster risk governance to manage disaster risk’ (UNDRR 2015). Embedded within this priority is the need to improve relevant laws and regulations and strengthen their implementation and enforcement (IFRC & UNDP 2015). Specifically, paragraph 27 (a) of the Sendai Framework states:

… to mainstream and integrate disaster risk reduction within and across all sectors and review and promote the coherence and further development, as appropriate, of national and local frameworks of laws, regulations and public policies … (UNDRR 2015:13)

This statement is important because policy direction and legal foundations assure the legitimacy of all the efforts to reduce the risks of disasters (UNISDR 2004). Legislation is critical in reducing disaster risks, addressing vulnerability factors and improving response to disasters to build safe and resilient communities. As Pelling and Holloway (2006) and Bang (2021) point out, disaster legislation is fundamental for protecting people and the environment. More importantly, laws define the priorities, institutional mandates, roles and responsibilities and other aspects of a national DRM system (IFRC & UNDP 2014).

According to Van Niekerk (2021), legal and statutory instruments guide action and ignite anticipatory governance in a bureaucratised environment such as in government. Legislation can empower agencies with new risk-reduction responsibilities or establish new institutions to undertake risk-reduction work (Pelling & Holloway 2006). As Toscano-Rivalta (2020) stated, legislating on disaster risk, defining concepts and establishing duties, responsibilities and accountabilities under the law may not be easy and may raise significant and complex questions, yet it must be done. Pelling and Holloway (2006) further state that legislative acts can be used to set budget lines and policy remits, which are crucial for risk reduction measures. Legislation is one of the several important instruments governments employ to organise and protect their citizens. In general, disaster management legislation is intended to guide the development of strategies for reducing disaster risks and improving response to build safe and resilient communities. Disaster legislation also guides risk reduction incorporation into development activities within a community (Bello, Bustamante & Pizarro 2021).

Meanwhile, as a global community, COVID-19 tested the integrity, strength and preparedness of societies, governments, communities and individuals to deal with the pandemic (Padayachee et al. 2020). Padayachee et al. (2020) further argue that neither developed nor developing countries were prepared for the impact of the COVID-19 pandemic. The global response to the COVID-19 pandemic was inadequate in most instances, especially regarding the institutional ability to deal with biological hazards. As a result, the pandemic reinforced and highlighted the need and urgency for multi-hazard risk assessments, unified responses and incident management in all linked systems (Van Niekerk 2021), all of which must be grounded in legislation. Fortunately, the DMA and NDMF, are clear on how they guide the reduction of disaster risks. Examples are the establishment of institutional arrangements, development of plans, funding, risk reduction strategies and many other guidelines.

### Implementation of the *Disaster Management Act* in response to COVID-19

The DMA provides for an integrated and coordinated policy that focuses on risk reduction and effective disaster response through the establishment of disaster management centres at national, provincial and municipal levels. Section 2 of the DMA states that the act does not apply to an occurrence that can be dealt with by other national legislation (Republic of South Africa 2002). In the COVID-19 case, the *National Health Act 61* of 2003 could not have been used because COVID-19 was a global concern, and there were more than 100 000 deaths.

As disaster management centres are branches within a line department, it indicates that they are considered a line function. It is, however, important to notice that DRM is a coordination function that should be at the highest level of decision-making backed by political will (Kunguma, Ncube & Mokhele 2021; Republic of South Africa 2005). According to Van Niekerk (2014), South Africa is one of the few countries that has yet to opt for placement of the disaster management function within the highest political office, that is the office of the President or Deputy President. The main argument for the placement of the National Centre in the highest political office is the need for decisive and mandated decisions on issues pertaining to hazards and disasters (Van Niekerk & Du Plessis 2020). The South Africa COVID-19 Country Report of 2021 argues that the powers and functions of the National Disaster Management Centre (NDMC) are, to some extent, restricted by its placement within Cooperative Governance and Traditional Affairs (CoGTA), which reduces its convening power (Republic of South Africa 2021).

After the World Health Organization (WHO) declared COVID-19 a global pandemic (WHO 2020), the President of South Africa was one of the first in Africa to declare COVID-19 a national disaster on 15 March 2020 (Kunguma et al. 2021; The Presidency 2020). Sections 3 and 27(1) of this act directed the responsible Minister in consultation with other Ministers to issue regulations in response to the disaster.

The declaration of a state of disaster is catered for in Section 27 of the act. However, the declaration is preceded by the classification of the disaster, which is performed by the Head of the Disaster Management Centre. Section 23 states:

When a disastrous event occurs or threatens to occur. the National Centre must, for the purpose of the proper application of this Act, determine whether the event should be regarded as a disaster in terms of this Act, and if so, the National Centre must immediately assess the magnitude and severity or potential magnitude and severity of the disaster; classify the disaster as a local, provincial, or national disaster in accordance with subsections (4), (5) and (6); and record the prescribed particulars concerning the disaster in the prescribed register … (Republic of South Africa 2002)

Once the disaster has been classified, Section 27 empowers the Minister to declare a state of disaster by Notice in the government gazette. This is if the existing legislation and contingency arrangements do not adequately provide for the national executive to deal effectively with the disaster or other special circumstances warrant the declaration of a national state of disaster. If a national state of disaster is declared in terms of subsection (1) the Minister may, subject to subsection (3), and after consulting the responsible Cabinet member, impose regulations or issue directions or authorise the issue of directions concerning amongst others the release of any available resources of the national Government including stores, equipment, vehicles and facilities (Nemakonde & Travis 2023).

The President and his cabinet members imposed a significant number of regulations and restrictions guided by the DMA to curb the spread of the virus. Cooperative Governance and Traditional Affairs and the Ministry of Health became the custodians of the COVID-19 pandemic hazard. Almost on a daily basis, these two custodians would brief the nation about statistics, regulations and other matters. Depending on the number of cases confirmed to be infected with the virus, a five-level COVID-19 alert system was introduced by the government to manage the easing of the lockdown and determine the level of restrictions to be applied (South African Government 2020):

- ‘Alert Level 1’ indicates a low COVID-19 spread with a high health system readiness;
- ‘Alert Level 2’ indicates a moderate COVID-19 spread with a high health system readiness;
- ‘Alert Level 3’ indicates a moderate COVID-19 spread with a moderate health system readiness;
- ‘Alert Level 4’ indicates a moderate to high COVID-19 spread with a low to moderate health system readiness; and
- ‘Alert Level 5’ indicates a high COVID-19 spread with a low health system readiness.

### A brief history of the South African disaster risk management legislation

In the late 1990s, South Africa began developing a unified and comprehensive legislation to deal with disasters (Republic of South Africa 2021), but the approach to disasters was mostly reactive. As in many other countries, many saw disasters as natural events that could be avoided. People believed we were at the mercy of disastrous events that could only be countered by providing relief and dealing with matters cascading from disaster impacts. At that time, the existing policies and institutions supported this thinking.

Following the severe flooding in the Western Cape in 1994 the country developed new legislative and policy frameworks for disaster management. The initial steps of the process involved a complete review of disaster management structures and policies (UNISDR 2004). As Pelling and Holloway (2006) point out, South Africa’s DRR legislation unfolded incrementally as part of a larger effort to reorient government institutions in the post-apartheid era. Over time, before the dawn of democracy and the Western Cape floods in 1994, several institutions were established, and legislation was promulgated to respond to disasters. The first institution was the Directorate for Emergency Planning (DEP), established in the Department of Justice in 1957 (Kunguma 2020; Sithole 2015). In 1963, the Directorate of Civil Defence replaced the DEP. In 1965, the Directorate was made independent by the Minister of Justice. Then, in 1966, the *Civil Defence Act 39* of 1966 was promulgated (later on repealed by the *Civil Protection Act 67* of 1977), and civil defence services were rendered at the national level only. In 1969, a Directorate of Civil Protection was instituted under the National Defence Force. As issues of humanitarian services require resources, the National Defence Force was a perfect fit for this purpose. Unfortunately, this institution’s function was military-focused and inappropriate for humanitarian assistance. They were not concerned with dealing with natural hazards or anthropomorphic hazards that could become disasters. Because of this gap, the *Civil Protection Act 67* of 1977 was revoked and replaced. This act promoted civil defence in all spheres of the Government, and the civil protection roles and response were delegated to either an appointed Chief Traffic Officer or Fireman (De Villiers Smit 1981; Kunguma 2020).

After realising the need to include natural hazards and increase resilience through better planning and strategies to increase community preparedness and other activities, the *Civil Protection Act* was amended in 1990. All references to ‘Civil Defence’ were amended to ‘Civil Protection’. All the changes that took place in the late 80s to early 90s coincided with changes that were happening internationally. Globally, in 1989, the International Decade for Natural Disaster Reduction (IDNR) declaration brought a paradigm shift from civil protection to disaster management. Change was now from a military-focused approach and the protection of the civilians mostly from war to responding to dealing with natural hazards.

In 1995, the cabinet recommended establishing a formal disaster management structure in the apartheid era. As a result, a National Disaster Management Committee was formed in 1996 and tasked with coordinating and managing disaster management policy (UNISDR 2004). However, this structure was never functional because there were no clear roles and responsibilities defined and no standard operating procedures put in place (Sithole 2015). As a result, an alternative structure, the Inter-Ministerial Committee for Disaster Management, was established in 1997. Ultimately, an interim disaster management authority was composed of representatives from 10 national departments, and this was later converted into a NDMC. An Inter-Departmental Disaster Management Committee (IDMC) was also established in 1999 to ensure better coordination among government departments at the national level.

Continuing with the disaster management paradigm shift, a Green Paper on Disaster Management was published in 1998. In 1999, a White Paper on Disaster Management was published within the framework of the IDNR. Following the White Paper, the DMA was promulgated in 2002 and the NDMF in 2005 as a framework for implementing the act. The adoption of the DMA ushered in a new era that shifted from traditional disaster response thinking to DRR, prevention and mitigation (Van Niekerk 2014). The act aims to provide for an integrated and coordinated disaster management policy that focuses on preventing or reducing the risk of disasters. It also aims to mitigate the severity of disasters, emergency preparedness rapid and effective response to disasters and post-disaster recovery. Establishing national, provincial and municipal disaster management centres and volunteers was also taken up (Republic of South Africa 2002). In 2015, the DMA was amended to Act 16 of 2015; one area it addresses is the call on the military and police services to assist in the event of a potential disaster (Section 7 of the *Amendment Act*). However, during the pandemic, there were reports of police and military brutality against civilians, leading to requests from the public to remove the military from responding to the pandemic (Bailie 2020). This amendment might require a call for further guidelines or standard operating procedures for the military and police during disasters and dealing with civilians.

## Research methods and design

This study aimed to analyse how South Africa’s DRM legislation was applied to respond to the COVID-19 pandemic. We conducted a content-based analysis of the *Disaster Risk Management Act* of 2002, grey literature and related peer reviewed scholarly literature. A qualitative content analysis of the legislative document, that is the South African DMA, peer-reviewed papers and grey literature (that is reports, opinion pieces and newspaper articles) was carried out. This is because we intended to understand how the application of the DRM legislation contributed to the response to the COVID-19 pandemic rather than generalising the findings from a study sample based on statistical inferences. Qualitative content analysis is a research technique for making replicable and valid inferences from texts (or other meaningful matters) to the context of their use (Krippendorff 2018). The content analysis examines data, printed matter, images or sound to understand what they mean to people, what they enable or prevent and what the information they convey means (Hall & Steiner 2020; Krippendorff 2004).

Whereas content analysis can be conducted both quantitatively or qualitatively or using mixed methods, qualitative analysis, was applied in this study (Bengtsson 2016). Specifically, conventional content analysis which is inductive in nature was applied in this study mainly because the knowledge on using DRM legislation to respond to COVID-19 is fragmented. Conventional content analysis mainly focuses on text data rather than counting the codes or key words. Forman and Damschroder (2007) believe that data generated inductively are categorised using categories derived from the data and, in most cases, applied to the data through close reading. A combination of latent analysis, which focuses on the interpretation of the underlying meaning of the text, and manifest content analysis which focuses on what the text says (Moldavska & Welo 2017), was applied to the study.

The corpus of our literature sample comprised English-written peer-reviewed papers, DRM legislation, newspaper articles, opinion pieces and reports. Whereas newspaper articles, opinion pieces and reports are not the main modes of communication amongst researchers, we consider them a unit of analysis mainly because a lot was published in these mediums in South Africa during the response to the COVID-19 pandemic. For peer-reviewed articles, computerised searches were conducted on well-established scientific databases (Scopus, Google Scholar and Web of Sciences). From a methodological perspective, selecting these databases represented the application of convenience sampling, a sample selected by the researcher by its convenient availability and practicability (Bryman & Bell 2015). Practically, we adopted the rationale that using a reduced number of representative search engines would facilitate the conduction of the study and the replicability of related outcomes in further research (Batista et al. 2018). In conducting a content-based literature review, we adopted steps outlined by Seuring and Gold (2012), which include:

- Material collection: Delimitation of the material and unit of analysis;
- Descriptive analysis: Initial descriptive analysis of the material;
- Category selection: Selection of the collected material according to specific analytic categories or dimensions;
- Material evaluation: Theoretically based analysis of the material according to the categories previously specified (Batista et al. 2018).

The documents were collected using internet-based searches. The inclusion criteria was that documents must focus South Africa’s disaster risk management policy and legislative frameworks, documents must link the use of legislation to address the COVID-19 pandemic. In this regard, the following key words were generated based on the focus and interest of the study and they were used individually and in combinations: DRM, South Africa, legislation, COVID-19, response, policy, frameworks and funding. These codes were further categorised into broad statements that were used to search for the relevant documents in the search engines.

Although this text was italicised in the manuscript it does not appear to be a quotation. Rather, it is a list of search statements that are reflected in Table 1.

**TABLE 1:** Data-collection methods of articles under review.

Search statements	Search results
Importance of disaster risk management legislation	122 000 000
SA DRM legislation and COVID-19	253 000
Funding for COVID-19 response in South Africa	110 000 000
Regulations for COVID-19 response under the DMA	66 400 000
South Africa’s disaster management legislative and policy frameworks	25 900 000
The extent to which the DRM legislation has been used to establish strong national structures for coordination during the COVID-19 response	76 000
Establishment of the National Coronavirus Command Council in South Africa	3 390 000

SA, South Africa; DRM, Disaster Risk Management; DMA, Disaster Management Act.

Therefore the recommendation is that this indented section is re-set in bullet points as stated in Table 1:

- Importance of disaster risk management legislation
- South African Disaster Risk Management legislation and COVID-19
- Funding for COVID-19 response in South Africa
- Regulations for COVID-19 response under the DMA
- South Africa’s disaster management legislative and policy frameworks
- The extent to which the DRM legislation has been used to establish strong national structures for coordination during the COVID-19 response
- Establishment of the National Coronavirus Command Council in South Africa

The choice of these statements was intentionally strict to narrow down the body of literature to contribute to the specific areas and aspects considered in this study. No other sampling criteria was added. Ultimately, 43 articles, reports, opinion pieces and newspaper articles were included in this review. This is not an exhaustive list of material available on the topic in South Africa, but we are satisfied that they are sufficient to address the study’s objective. The overall results of the search are reflected in Table 1.

The content analysis of the collected document was carried out using a process that involves several steps including the development of key statements instead of key words, manual analysis of the documents, which involves reading documents thoroughly to understand the context in which the statements were used. The findings of this analysis are presented in the next section using the broad themes that were generated during the reading of the documents. The presentation of the findings using the themes draws heavily from Bengtsson (2016) who opines that in qualitative content analysis, data are presented in words and themes as opposed to frequencies expressed as a percentage or actual number of key categories. He (Bengtsson 2016) argues that this makes it possible to draw some interpretations of the result rather than summarising the results.

### Ethical considerations

This article does not contain any studies involving human participants performed by any of the authors.

## Results

### DRM legislation to establish strong national COVID-19 coordination structures

The COVID-19 exposed the need for the NDMC in South Africa to be placed in government functions with high decision-making powers such as the Presidency for the national office (Van Niekerk & Du Plessis 2020). The South Africa COVID-19 Country Report of 2021 argues that the mandate and role of Disaster Management Centres were not clearly understood during the response to COVID-19, and it was sometimes difficult to establish relationships with the Department of Health to coordinate with the national response because they are both at the same level of decision-making powers (Republic of South Africa 2021). For Van Niekerk (2014), the organising, leading, control, funding provision, coordination and implementation of the DMA and NDMF are all constrained by the placement of the NDMC in a line ministry. During the response to the pandemic, the location of the NDMC led to contradictory statements made by the Minister responsible for the Department and the President of the country. There were also contradictions with other members of the Cabinet (Padayachee et al. 2020; Singh 2023). One such contradiction was the banning and unbanning of cigarette sales; the President announced the end of the ban on 01 May 2020, and the Minister of CoGTA announced that the ban would not be lifted (De Vos 2020; Kunguma et al. 2021). Therefore, if the disaster management function were in the highest decision-making office, there would be one voice and fewer contradictions, this is because the Minister in the Presidency would have these responsibilities (Van Niekerk & Du Plessis 2020).

### South Africa disaster risk management legislation and COVID-19

Both the classification and the declaration of the state of disaster for COVID-19 were published on Government Notice Number 43096 of 15 March 2020 (Department of Cooperative Governance and Traditional Affair 2020a). As a result of this declaration, the President of South Africa announced a 21-day country lockdown on 26 March 2020. The act makes provision for the state of disaster to remain active for 3 months. The act states in Section 27(5), that if it (a); lapses 3 months after it has been declared, (b) it may be terminated (Republic of South Africa 2002). The Minister communicates the termination by means of a notice in the Gazette before it lapses in terms of sub-section (a). According to subsection (c), the state of disaster may also be extended by the Minister by notice in the Gazette for 1 month before it lapses or the existing extension is due to expire. The challenge with this subsection is that it needs to state how long a disaster can be extended.

The 3 months prescribed in the DMA proved impractical with the COVID-19 pandemic because the virus evolved and different strains emerged while vaccines were not forthcoming. Although it was necessary to remain in a state of disaster to combat the virus, the regulations imposed brought many social and economic challenges. In this regard, the social and economic challenges brought on by the state of disaster regulations, such as lockdown, caused a rise in gender-based violence, loss of jobs, closure of businesses, disruption of service delivery like water supply, and stoppage of school, amongst others. For 2 years, businesses, civil society and opposition parties tried to fight and call for the end of the state of disaster. However, an earlier end did not occur because it is assumed that the Cabinet needed help finding other legislation or organs of state-controlled contingencies to manage the pandemic (Harper 2022).

### Regulations for COVID-19 response under the *Disaster Management Act*

After the declaration of the state of disaster, regulations were issued in terms of Section 27(2) of the act, which were published in Government Notice no. 43107 of 18 March 2020 (Department of Cooperative Governance and Traditional Affairs 2020b). These regulations were amended as it became necessary (Department of Cooperative Governance and Traditional Affairs 2020c). South Africa went into a nationwide lockdown on 26 March 2020, which was extended through a risk-adjusted approach ranging from Alert Level 5 to Alert Level 1 (Department of Cooperative Governance and Traditional Affairs 2020d). The changing warning levels and policies instituted by the government in response to the pandemic’s ever-changing nature helped to facilitate and led to greater compliance by members of the public (Enaifoghe 2021).

Broadly speaking, the regulations covered the release of resources, prevention and prohibition of gatherings, refusal of medical examination, prophylaxis, isolation and quarantine, places of quarantine and isolation, closure of schools and partial care facilities, limitations on the sale dispensing or transportation of liquor and offences and penalties among others. These regulations shut down the economy, disrupting social lives as people were confined to their homes, essentially limiting their civil rights and liberties. The South African National Defence Force was deployed in various cities nationwide to enforce the lockdown restrictions. The deployment of the army to enforce the lockdown in South Africa was criticised from a human rights perspective and fears of brutality were compounded by evidence from across the country (Singh & Tembo 2022).

Some decisions and regulations were found by the courts to be irrational and not justifiable and other cases dealt with the constitutionality of the regulations based on various grounds (Singh & Tembo 2022).

### Establishment of the National Coronavirus Command Council in South Africa

Establishing and using structures not provided for in the DRM legislation came under heavy criticism. South Africa’s COVID-19 Country Report of 2021 points out that the legislative framework used to create these structures needed to be clarified, some of which appeared to duplicate the national, provincial and local disaster management centres. Sections 4 and 5 of the act establish two important intergovernmental structures to deal with all issues pertaining to disasters in South Africa: the Intergovernmental Committee on Disaster Risk Management (ICDM) and the National Disaster Management Advisory Forum (NDMAF). The ICDM should consist of Cabinet Members, Members of Executive Committees (MEC) from each province, and members of municipal councils. The committee should be chaired by the Minister responsible for the portfolio. On the other hand, the NDMAF should include senior representatives from each national department whose Minister is a member of the ICDM, as well as senior representatives from each provincial department whose MEC is a member of the ICDM. Additionally, municipal officials selected by the South African Local Government Association (SALGA) and representatives of other disaster management stakeholders should be included. The Head of the NDMC must chair the NDMAF. In response to the COVID-19 pandemic, these two structures were not activated. This was because the two structures were not functional before the pandemic occurred. In any way, Van Niekerk argued in 2014 that the structures provided for the DMA need to be productive. This non-functionality of the structures was acknowledged in the South Africa COVID-19 Country Report of 2021, which the ICDM had not met for many years (Republic of South Africa 2021). Van Niekerk and Du Plessis (2020) are more precise, stating that the ICDM has not had a meeting since 2002. This means that the committee was established but was never in existence.

Instead of the structures provided for in the act, several structures were established to effectively coordinate the response to COVID-19. The National Coronavirus Command Council (NCCC) was established and comprised of Cabinet Ministers from portfolios impacted by COVID-19, including the Departments of Health, Justice, Home Affairs, Defence Force and the Police. The structure was established as a committee of cabinet. This structure was expanded to include all cabinet ministers and was chaired by the President. In his parliamentary reply to a question by one of Parliament members, the President acknowledged that the NCCC was not established in terms of the DMA. The President also responded ‘NO’ to questions on whether the NCCC has replaced or was duplicating roles, responsibilities and functions of the structures provided in the act, that is ICDM and NDMAF. The public needed to understand why the ICDM, which is constitutional and was not used in the DMA, was not used (Van Niekerk & Du Plessis 2020). The President indicated that:

[*T*]he NCCC coordinates the Government’s response to the coronavirus pandemic. The NCCC recommends measures required in terms of the national state of disaster to the Cabinet. Cabinet makes the final decisions. (Merten 2020:1)

However, the Supreme Court of Appeal ruled that the NCCC was lawful and constitutional as a cabinet committee. As the disaster was a health hazard, the *South African National Health Act, 61* of 2003 could have been used. The act empowers the health minister to appoint an advisory and technical committee (Singh 2023). In partnership with CoGTA, the Department of Health could have established such a constitutional body, but government officials characterised the Health Act as inadequate to deal with COVID-19 (Karrim 2020).

The National Joint Operational and Intelligence Structure (NatJOINTS), which comprised senior public servants from the national and provincial governments, was activated to provide ongoing coordination. National Joint Operational and Intelligence Structure provided a platform for coordination between the sectors and helped to ensure implementation plans were implemented, as captured in the response plan. The NDMC assembled a network of experts that could help coordinate the response to any future disasters. According to Flanagan (2016), citing the Minister of Police, the NatJOINTS is responsible for developing and implementing operational safety plans. They also provide a safe and secure environment at big events, prioritising peace and stability of the country and handling issues of immigration and maritime security. However, the NatJOINTS were perceived as passive, playing the role of advice and information provision only. As the South African Police Services chairs the NatJOINTS, it is viewed as though the institutional arrangement is militaristic (United Nations Development Programme & Department of Cooperative Governance and Traditional Affairs 2021).

### Funding for COVID-19 response in South Africa

Section 56. (1) of the DMA states that ‘the chapter is subject to Sections 16 and 25 of the *Public Finance Management Act* (PFMA), 1999’, which provides for using funds in emergencies at both national and provincial levels. Blecher et al. (2021) point out that within the South African PFM framework, there is a range of mechanisms to respond to emergencies and other unforeseen events. Several of these were used in the COVID-19 response. As such funding for response to COVID-19 is one area that received much praise. According to Rahim et al. (2020), many countries reprogrammed their existing budgets, activated contingency reserves, and adopted supplementary budgets in response to the COVID-19 pandemic. To further mobilise resources and accelerate emergency spending, many countries also created dedicated COVID-19 extrabudgetary funds (EBFs) (Rahim et al. 2020). In South Africa, the Provincial Disaster Relief Grant, a conditional grant managed by the Department of Co-operative Governance and Traditional Affairs, was used to allocate R466 million to Provincial Health Departments to fund initial PPE needs (Blecher et al. 2021). Subsequently, various departments approved or reallocated additional funds for PPE and other expenses. The National Treasury issued formal guidance to all departments to use provisions in Section 29 of the PFMA (1 of 1999) to start spending immediately at the start of the financial year and approved the reallocation of funds within departments when needed. Importantly, procurement rules were eased to enable the rapid purchase of PPE, but after abuse of these funds, these amendments were eventually revoked (Blecher et al. 2021). Thirdly, the Special Adjustments Budget (SAB) tabling in June 2020 was the most comprehensive budgetary intervention. The sources of funding are reflected in Table 2.

**TABLE 2:** Funding sources for the COVID-19 fiscal response package.

Sources	*R* million
Credit guarantee scheme	200 000
Baseline reprioritisation	130 000
Borrowings from multilateral finance institutions and development banks^a^ for business support, job creation and protection	95 000
Additional transfers and subsidies from the social security funds	60 000
Available funds in the Department of Social Development 2020/21 appropriation	15 000
**Total**	**500 000**

*Source*: National Treasury, 2021, *Economic measures for COVID-19*, Department of Republic of South Africa

a , International Monetary Fund, World Bank and the New Development Bank.

More importantly, South Africa’s Government rolled out a social relief and economic support package worth R500 billion, approximately 10% of GDP (Noyoo 2021). This package funded, for instance, a special COVID-19 ‘Social Relief of Distress Grant’ for all those individuals who were unemployed and did not receive any other form of social assistance (Noyoo 2021). Thus, a special COVID-19 ‘Social Relief of Distress Grant’ of ZAR 350 a month was paid out to individuals who were unemployed and did not receive any other form of social grant. At the expiry of the social grant system, the South African Government expanded the system through a short-term 6-month heterogeneous increase in all existing social grants (Baskaran, Bhorat & Köhler 2020; Department of Social Development 2021). It is estimated that the ‘COVID-19 social assistance package’ had the potential to reach 36 million people or 63% of the South African population (Baskaran et al. 2020; Department of Social Development 2021). South Africa’s COVID-19 fiscal response package is reflected in Table 3.

**TABLE 3:** The COVID-19 fiscal response package.

Support package	*R* million
Credit guarantee scheme	200 000
Job creation and support for SME and informal business	100 000
Measures for income support (Further tax deferrals, SDL holiday and ETI extension)	70 000
Support to vulnerable households for 6 months	50 000
Wage protection (UIF)	40 000
Health and other frontline services	20 000
Support to municipalities	20 000
**Total**	**500 000**

*Source*: National Treasury, 2021, *Economic measures for COVID-19*, Department of Republic of South Africa

SME, small and medium-sized enterprises; SDL, skills development levy; ETI, employment tax incentive; UIF, unemployment insurance fund.

The budget provision for the health response to COVID-19 exceeded R20 billion, which was achieved through additional allocations and reprioritisation, while income protection measures exceeded R100 billion (Blecher et al. 2021). Other government social policy interventions related to tax relief measures, relief funds, emergency procurements, wage support through the UIF and funding to small businesses were applied (Noyoo 2021). The Solidarity Fund stood out as a successful partnership between government, social partners, and academia working together to ensure national resilience and hope. In addition, in order to respond to the immediate needs of vulnerable citizens, the Department of Social Development partnered with the Solidarity Fund, non-governmental organisations (NGOs) and community-based organisations (CBOs) to distribute 250 000 food parcels across the country (Noyoo 2021).

## Conclusion

This article evaluated the strengths and shortcomings of South Africa’s DRM legislation in guiding the response to the COVID-19 pandemic. Whereas there were some positive outcomes resulting from the use of the DMA, 2002 to guide response to the COVID-19 pandemic, the study identified significant shortcomings in the South African DRM legislation, and these include the placement of the NDMC, the establishment of new structures for COVID-19 response and poor regulations that were found to be irrational and not justifiable by the courts. Also, whereas the legislation has been hailed as one of the most progressive DRM legislation in the 21st century, the legislation was never previously applied to any situation involving biological hazards such as pandemics. This could explain the deficiencies that came with the use of the legislation. In lieu of the challenges faced in applying the DMA in response to COVID-19, we agree with Padayachee et al. (2020) that South Africa needs to rethink its disaster management policies, guidelines and strategic frameworks. We argue that COVID-19 provided opportunities to reinstitute and better the structures provided in the act to make them functional. Therefore, we recommend the amendment of the DMA based on the experience during the COVID-19 pandemic to respond appropriately to future disasters. We contend that the amended legislation must be anticipatory to provide better planning for better response. We, however, submit that improving the DRM system will not stop hazards such as COVID-19 from happening but will rather reduce the impacts of the impending disasters on the lives, health, infrastructure and livelihoods of communities. Future research can focus on applying the DMA to declare a state of disaster to other special circumstances, such as the energy crisis.
